# Supplementary material for: Artificial Intelligence Predictive Models of Response to Cytotoxic Chemotherapy Alone or Combined to Targeted Therapy for Metastatic Colorectal Cancer Patients: A Systematic Review and Meta-Analysis
Source: Cancers (Basel). 2022 Aug 19;14(16):4012. doi: 10.3390/cancers14164012 (PMC9406544; doi:10.3390/cancers14164012)
Supplement: Supplementary file 1 [file cancers-14-04012-s001.zip › cancers-1814972-supplementary.pdf]

Supplemental Table S1. Study quality of eligible publications, modified Luo scale [71]

Section	Checklist item	Abraham et al. 2021	Barat et al. 2020	Chen et al. 2014	Defeudis et al. 2021	Del Rio et al. 2007	Dercle et al. 2020	Giannini et al. 2022	Giannini et al. 2020	Granata et al. 2021	Johnson et al. 2022	Li et al. 2022	Liu et al. 2022
Title	Identify the report as introducing a model	Yes	No	Yes	Yes	Yes	Yes	Yes	Yes	Yes	Yes	Yes	Yes
Abstract	Background	Yes	Yes	Yes	No	No	No	Yes	No	Yes	Yes	Yes	Yes
	Objectives	Yes	Yes	Yes	Yes	Yes	Yes	Yes	Yes	Yes	Yes	Yes	Yes
	Data sources	Yes	Yes	No	No	Yes	Yes	Yes	Yes	Yes	Yes	Yes	Yes
	Machine learning methods applied	Yes	Yes	Yes	Yes	No	No	Yes	Yes	Yes	Yes	Yes	Yes
	Key result(s)	Yes	Yes	Yes	Yes	Yes	Yes	Yes	Yes	Yes	Yes	Yes	Yes
	Conclusion including the practical value of the model	Yes	Yes	Yes	Yes	Yes	Yes	Yes	Yes	Yes	Yes	Yes	Yes
Intro-duction	Identify the clinical goal	Yes	Yes	Yes	Yes	Yes	Yes	Yes	Yes	Yes	Yes	Yes	Yes
	Review the current practice and existing models	Yes	Yes	Yes	No	Yes	Yes	Yes	Yes	Yes	Yes	Yes	Yes

	State the nature of study as being a model based on machine learning methods	Yes	Yes	Yes	No	No	Yes	Yes	Yes	No	Yes	Yes	Yes
	Identify how the study may benefit the clinical goal	Yes	Yes	Yes	Yes	No	Yes	Yes	Yes	Yes	Yes	Yes	Yes
Methods	Identify the clinical setting for the target model.	Yes	Yes	Yes	Yes	Yes	Yes	Yes	Yes	Yes	Yes	Yes	Yes
	Specify if the study is retrospective or prospective.	Yes	Yes	Yes	Yes	Yes	Yes	Yes	Yes	Yes	Yes	Yes	Yes
	Specify if the model is prognostic or diagnostic	Yes	Yes	Yes	Yes	Yes	Yes	Yes	Yes	Yes	Yes	Yes	Yes
	Identify relevant data sources and quote the ethics approval for data access.	Yes	Yes	Yes	Yes	Yes	Yes	Yes	Yes	Yes	Yes	Yes	Yes
	State the inclusion and exclusion criteria for data.	Yes	No	No	No	Yes	No	Yes	Yes	Yes	Yes	Yes	No
	Describe the time span of data and the sample or cohort size.	Yes	Yes	Yes	Yes	Yes	Yes	Yes	Yes	Yes	Yes	Yes	Yes
	Define the variables	Yes	Yes	Yes	Yes	Yes	Yes	Yes	Yes	Yes	Yes	Yes	Yes

	Describe the basic statistics of the dataset, particularly of the response variable.	Yes	Yes	Yes	Yes	Yes	Yes	Yes	Yes	Yes	Yes	Yes	Yes
	Define how outcomes are measured	Yes	Yes	Yes	Yes	Yes	Yes	Yes	Yes	Yes	Yes	Yes	Yes
	Determine the form of the model (e.g., classification, regression).	Yes	Yes	Yes	Yes	Yes	Yes	Yes	Yes	Yes	Yes	Yes	Yes
	Define the success criteria for prediction	Yes	Yes	No	Yes	Yes	Yes	Yes	No	Yes	Yes	Yes	Yes
	Specify the modeling techniques. If only one type of model was used, justify the decision for using that model.	Yes	Yes	Yes	Yes	Yes	Yes	Yes	Yes	Yes	Yes	Yes	Yes
	State if outliers with impossible or extreme responses are removed; state any criteria used for outlier removal.	No	No	No	Yes	No	No	Yes	Yes	Yes	Yes	Yes	No
	State how missing values were handled.	No	No	No	No	No	Yes	Yes	Yes	Yes	Yes	Yes	No
	Split the data into a derivation/training set and a validation/ test set.	Yes	Yes	Yes	Yes	No	Yes	Yes	Yes	Yes	Yes	Yes	Yes

	Specify the model validation strategies	Yes	Yes	Yes	Yes	Yes	Yes	Yes	Yes	Yes	Yes	Yes	Yes
	Implement the model selection strategy	No	No	No	No	Yes	Yes	Yes	Yes	Yes	Yes	Yes	Yes
	External validation should also be performed whenever possible.	Yes	Yes	No	No	No	No	Yes	No	Yes	Yes	Yes	Yes
	Report the validation metrics.	Yes	Yes	Yes	Yes	Yes	Yes	Yes	Yes	Yes	Yes	Yes	Yes
Results	Assess whether sufficient data were available for a good fit of the model	Yes	Yes	Yes	Yes	Yes	Yes	Yes	Yes	Yes	Yes	Yes	Yes
	Report the predictive performance of the final model in terms of the validation metrics specified in the methods section.	Yes	Yes	Yes	Yes	Yes	Yes	Yes	Yes	Yes	Yes	Yes	Yes
	If possible, report the parameter estimates in the model and their confidence intervals or report non-parametric estimates from bootstrap samples.	Yes	Yes	Yes	No	Yes	Yes	Yes	No	Yes	No	Yes	No
	If possible, report what variables were shown to be predictive/prognostic	Yes	Yes	Yes	Yes	Yes	Yes	Yes	Yes	Yes	Yes	Yes	Yes

	of the outcome variable.												
	Report findings from internal/ external validation	Yes	Yes	Yes	Yes	Yes	Yes	Yes	Yes	Yes	Yes	Yes	Yes
Discus-sion	Interpretation of the final model.	Yes	Yes	Yes	Yes	Yes	Yes	Yes	Yes	Yes	Yes	Yes	Yes
	Report the clinical implications derived from the obtained predictive performance.	Yes	Yes	Yes	Yes	Yes	Yes	Yes	Yes	Yes	Yes	Yes	Yes
	Include discussion on (1) balance between model accuracy and model simplicity or interpretability, and (2) the familiarity with the modeling techniques of the end user.	No	No	No	No	No	Yes	Yes	Yes	Yes	Yes	Yes	Yes
	Discuss model in context of other models in the literature	Yes	Yes	Yes	No	Yes	Yes	Yes	Yes	Yes	Yes	Yes	Yes
	Potential pitfalls in interpreting the model	No	No	No	No	No	No	Yes	Yes	Yes	Yes	Yes	Yes
	Potential bias of the data used in modeling	Yes	No	No	No	No	Yes	Yes	Yes	No	Yes	Yes	Yes

	Generalizability of the data	No	Yes	No	Yes	No	No	Yes	No	No	No	Yes	Yes

Section	Checklist item	Lu et al. 2021	Lu et al. 2020	Maaref et al. 2020	Nakanishi et al. 2021	Naseem et al. 2021	Oyaga-Iriarte et al. 2019	Tian et al. 2021	Tsuji et al. 2012	Ubels et al. 2020	Vera-Yunca et al. 2020	Wei et al. 2021
Title	Identify the report as introducing a model	Yes	Yes	Yes	Yes	Yes	Yes	Yes	Yes	Yes	Yes	Yes
Abstract	Background	Yes	Yes	Yes	Yes	Yes	Yes	Yes	Yes	Yes	Yes	No
	Objectives	Yes	Yes	Yes	Yes	Yes	Yes	Yes	Yes	Yes	Yes	Yes
	Data sources	Yes	Yes	Yes	Yes	Yes	Yes	Yes	Yes	Yes	Yes	Yes
	Machine learning methods applied	Yes	Yes	Yes	Yes	Yes	Yes	Yes	Yes	Yes	Yes	Yes
	Key result(s)	Yes	Yes	Yes	Yes	Yes	Yes	Yes	Yes	Yes	Yes	Yes
	Conclusion including the practical value of the model	Yes	Yes	Yes	Yes	Yes	Yes	Yes	Yes	Yes	Yes	Yes
Intro-duction	Identify the clinical goal	Yes	Yes	Yes	Yes	Yes	Yes	Yes	Yes	Yes	Yes	Yes
	Review the current practice and existing models	Yes	Yes	Yes	Yes	Yes	Yes	Yes	Yes	Yes	Yes	Yes
	State the nature of study as being a model based on	Yes	Yes	Yes	Yes	Yes	Yes	Yes	Yes	Yes	Yes	Yes

	machine learning methods											
	Identify how the study may benefit the clinical goal	Yes	Yes	Yes	Yes	Yes	Yes	Yes	Yes	Yes	Yes	Yes
Methods	Identify the clinical setting for the target model.	Yes	Yes	Yes	Yes	Yes	Yes	Yes	Yes	Yes	Yes	Yes
	Specify if the study is retrospective or prospective.	Yes	Yes	Yes	Yes	Yes	Yes	Yes	Yes	Yes	Yes	Yes
	Specify if the model is prognostic or diagnostic	Yes	Yes	Yes	Yes	Yes	Yes	Yes	Yes	Yes	Yes	Yes
	Identify relevant data sources and quote the ethics approval for data access.	Yes	Yes	Yes	Yes	Yes	Yes	Yes	Yes	Yes	Yes	Yes
	State the inclusion and exclusion criteria for data.	Yes	Yes	Yes	Yes	Yes	Yes	Yes	Yes	Yes	Yes	Yes
	Describe the time span of data and the sample or cohort size.	Yes	Yes	Yes	Yes	Yes	Yes	Yes	Yes	Yes	Yes	Yes
	Define the variables	Yes	Yes	Yes	Yes	Yes	Yes	Yes	Yes	Yes	Yes	Yes

	Describe the basic statistics of the dataset, particularly of the response variable.	Yes	Yes	Yes	Yes	Yes	Yes	Yes	Yes	Yes	Yes	Yes
	Define how outcomes are measured	Yes	Yes	Yes	Yes	Yes	Yes	Yes	Yes	Yes	Yes	Yes
	Determine the form of the model (e.g., classification, regression).	Yes	Yes	Yes	Yes	Yes	Yes	Yes	Yes	Yes	Yes	Yes
	Define the success criteria for prediction	Yes	Yes	Yes	Yes	Yes	Yes	Yes	Yes	Yes	Yes	Yes
	Specify the modeling techniques. If only one type of model was used, justify the decision for using that model.	Yes	Yes	Yes	Yes	Yes	Yes	Yes	Yes	Yes	Yes	Yes
	State if outliers with impossible or extreme responses are removed; state any criteria used for outlier removal.	No	No	No	Yes	No	No	Yes	Yes	No	Yes	No
	State how missing values were handled.	No	No	No	Yes	No	No	Yes	Yes	No	No	No
	Split the data into a derivation/training	Yes	Yes	Yes	No	Yes	No	Yes	Yes	Yes	Yes	Yes

	set and a validation/ test set.											
	Specify the model validation strategies	Yes	Yes	Yes	Yes	Yes	Yes	Yes	Yes	Yes	Yes	Yes
	Implement the model selection strategy	Yes	Yes	Yes	Yes	Yes	Yes	Yes	Yes	Yes	Yes	Yes
	External validation should also be performed whenever possible.	No	No	No	No	Yes	No	Yes	No	No	No	No
	Report the validation metrics.	Yes	Yes	Yes	Yes	Yes	Yes	Yes	Yes	Yes	Yes	Yes
Results	Assess whether sufficient data were available for a good fit of the model	Yes	Yes	Yes	Yes	Yes	Yes	Yes	Yes	Yes	Yes	Yes
	Report the predictive performance of the final model in terms of the validation metrics specified in the methods section.	Yes	Yes	Yes	Yes	Yes	Yes	Yes	Yes	Yes	Yes	Yes
	If possible, report the parameter estimates in the model and their confidence intervals or report non-parametric estimates from bootstrap samples.	Yes	Yes	Yes	Yes	No	No	Yes	No	Yes	Yes	No

	If possible, report what variables were shown to be predictive/prognostic of the outcome variable.	Yes	Yes	Yes	Yes	Yes	Yes	Yes	Yes	Yes	Yes	Yes
	Report findings from internal/ external validation	Yes	Yes	Yes	Yes	Yes	Yes	Yes	Yes	Yes	Yes	Yes
Discus-sion	Interpretation of the final model.	Yes	Yes	Yes	Yes	Yes	Yes	Yes	Yes	Yes	Yes	Yes
	Report the clinical implications derived from the obtained predictive performance.	Yes	Yes	Yes	Yes	Yes	Yes	Yes	Yes	Yes	Yes	Yes
	Include discussion on (1) balance between model accuracy and model simplicity or interpretability, and (2) the familiarity with the modeling techniques of the end user.	Yes	Yes	No	Yes	No	No	Yes	Yes	Yes	Yes	Yes
	Discuss model in context of other models in the literature	Yes	Yes	Yes	Yes	Yes	Yes	Yes	Yes	Yes	Yes	Yes

	Potential pitfalls in interpreting the model	Yes	Yes	Yes	Yes	Yes	Yes	Yes	Yes	Yes	Yes	Yes
	Potential bias of the data used in modeling	Yes	Yes	Yes	Yes	Yes	Yes	Yes	Yes	Yes	Yes	No
	Generalizability of the data	Yes	No	No	Yes	Yes	No	Yes	Yes	Yes	Yes	Yes

Section	Checklist item	Williams et al. 2021	Yuan et al. 2012	Zhu et al. 2020
Title	Identify the report as introducing a model	Yes	Yes	Yes
Abstract	Background	Yes	Yes	Yes
	Objectives	Yes	Yes	Yes
	Data sources	Yes	Yes	Yes
	Machine learning methods applied	Yes	Yes	Yes
	Key result(s)	Yes	Yes	Yes
	Conclusion including the practical value of the model	Yes	Yes	Yes
Intro-duction	Identify the clinical goal	Yes	Yes	Yes
	Review the current practice and existing models	Yes	Yes	Yes
	State the nature of study as being a model based on machine learning methods	Yes	Yes	Yes

	Identify how the study may benefit the clinical goal	Yes	Yes	Yes
Methods	Identify the clinical setting for the target model.	Yes	Yes	Yes
	Specify if the study is retrospective or prospective.	Yes	Yes	Yes
	Specify if the model is prognostic or diagnostic	Yes	Yes	Yes
	Identify relevant data sources and quote the ethics approval for data access.	Yes	Yes	Yes
	State the inclusion and exclusion criteria for data.	Yes	Yes	Yes
	Describe the time span of data and the sample or cohort size.	Yes	Yes	Yes
	Define the variables	Yes	Yes	Yes
	Describe the basic statistics of the dataset, particularly of the response variable.	Yes	Yes	Yes

	Define how outcomes are measured	Yes	Yes	Yes
	Determine the form of the model (e.g., classification, regression).	Yes	Yes	Yes
	Define the success criteria for prediction	Yes	Yes	Yes
	Specify the modeling techniques. If only one type of model was used, justify the decision for using that model.	Yes	Yes	Yes
	State if outliers with impossible or extreme responses are removed; state any criteria used for outlier removal.	No	No	No
	State how missing values were handled.	No	No	Yes
	Split the data into a derivation/training set and a validation/ test set.	Yes	Yes	Yes
	Specify the model validation strategies	Yes	Yes	Yes
	Implement the model selection strategy	Yes	Yes	Yes

	External validation should also be performed whenever possible.	No	No	Yes
	Report the validation metrics.	Yes	Yes	Yes
Results	Assess whether sufficient data were available for a good fit of the model	Yes	No	Yes
	Report the predictive performance of the final model in terms of the validation metrics specified in the methods section.	Yes	Yes	Yes
	If possible, report the parameter estimates in the model and their confidence intervals or report non-parametric estimates from bootstrap samples.	Yes	No	Yes
	If possible, report what variables were shown to be predictive/prognostic of the outcome variable.	No	Yes	Yes
	Report findings from internal/ external validation	Yes	Yes	Yes
Discus-sion	Interpretation of the final model.	Yes	Yes	Yes

	Report the clinical implications derived from the obtained predictive performance.	Yes	Yes	Yes
	Include discussion on (1) balance between model accuracy and model simplicity or interpretability, and (2) the familiarity with the modeling techniques of the end user.	Yes	Yes	Yes
	Discuss model in context of other models in the literature	Yes	Yes	Yes
	Potential pitfalls in interpreting the model	Yes	No	Yes
	Potential bias of the data used in modeling	No	No	No
	Generalizability of the data	Yes	No	Yes
